# Approaching the strong coupling limit in single plasmonic nanorods interacting with J-aggregates

**DOI:** 10.1038/srep03074

**Published:** 2013-10-29

**Authors:** Gülis Zengin, Göran Johansson, Peter Johansson, Tomasz J. Antosiewicz, Mikael Käll, Timur Shegai

**Affiliations:** 1 Department of Applied Physics, Chalmers University of Technology, 412 96 Göteborg, Sweden; 2 Department of Microtechnology and Nanoscience, Chalmers University of Technology, 412 96 Göteborg, Sweden; 3 School of Science and Technology, Örebro University, 701 82 Örebro, Sweden; 4 Centre of New Technologies, University of Warsaw, Zwirki i Wigury 93, 02-089 Warsaw, Poland

## Abstract

We studied scattering and extinction of individual silver nanorods coupled to the J-aggregate form of the cyanine dye TDBC as a function of plasmon – exciton detuning. The measured single particle spectra exhibited a strongly suppressed scattering and extinction rate at wavelengths corresponding to the J-aggregate absorption band, signaling strong interaction between the localized surface plasmon of the metal core and the exciton of the surrounding molecular shell. In the context of strong coupling theory, the observed “transparency dips” correspond to an average vacuum Rabi splitting of the order of 100 meV, which approaches the plasmon dephasing rate and, thereby, the strong coupling limit for the smallest investigated particles. These findings could pave the way towards ultra-strong light-matter interaction on the nanoscale and active plasmonic devices operating at room temperature.

Plasmonic cavities and optical antennas possess a number of properties that could be crucial to the development of active nanophotonic devices. Examples of advantages include the nanoscale footprint, broadband room-temperature operation and strong local electromagnetic field amplification^1^. On the other hand, plasmonic nanostructures exhibit relatively low resonance quality factors (Q < 100) and they typically possess only weak nonlinearities, thereby essentially prohibiting saturation and active control. One way to overcome this problem could be to combine plasmonic structures with dyes, quantum dots, nitrogen vacancy centers or other quantum light sources that combine narrow electronic resonances and high oscillator strengths with a possibility for active manipulation through, for example, doping, optical saturation or external fields.

Recent theoretical predictions of electromagnetically-induced transparency (EIT) and vacuum Rabi splitting in dimer nanoantennas loaded with single quantum dots positioned at antenna “hot-spots”^2^,^3^,^4^,^5^ have indicated exciting possibilities for room-temperature cavity quantum electrodynamics (cQED) and quantum optics applications. Similarly, strong light-matter interactions have also been predicted for surface excitations in patterned graphene nanostructures^6^ and recent experimental reports include observations of super-quenching^7^ and single-photon optical transistor effects in single-molecules at low temperature^8^ and theoretically investigated quantum emitter strongly coupled to thin metallic wires^9^.

True strong coupling implies a coupling stronger than the dissipative broadening of both the emitter and the cavity. The strong coupling regime allows for studies of fundamental aspects of quantum mechanics, such as entanglement and decoherence processes, as well as for exploration of potential applications, like photonic quantum communication and quantum information processing. Strong coupling at optical frequencies was first observed for atoms passing through a high finesse Fabry Perot etalon^10^ and recently demonstrated in the solid state using single quantum dots coupled to ultra-high quality factor photonic crystal^11^,^12^ and micropillar cavities^13^. For small cavity mode volumes and large dipole moments of the quantum dot, the dot-cavity coupling strength can be made as strong as 30 GHz^14^, which exceeds the decay rate of the cavity mode while still requiring the experiment to be carried out at cryogenic temperatures in order to have a sufficiently low quantum dot dephasing rate. In plasmonics, the effective mode volume can be reduced further, thus pushing the coupling strength towards the THz regime and possible room temperature operation. Experimental reports of strong coupling between surface plasmons and excitons formed in large ensembles of adsorbed molecules, in molecular aggregates or in quantum dots, include studies of propagating surface plasmons in thin metal films^15^,^16^,^17^,^18^,^19^, localized plasmons in hole and particle arrays^20^,^21^,^22^,^23^,^24^, ensembles of various nanoparticles^25^,^26^,^27^,^28^,^29^,^30^,^31^, surface-enhanced Raman scattering (SERS)^29^,^31^ and low quality factor Fabry-Perot cavities^32^,^33^. However, these observations were mainly made on the ensemble level and reports on the single nanoparticle level are relatively few^34^,^35^,^36^,^37^.

One of the first single particle studies was dedicated to the interaction between a cyanine dye and gold nanoparticles^34^. Later, rather distinct molecular absorption features, interpreted as “plasmon resonance energy transfer”, were observed in the scattering spectra of plasmonic nanoparticles covered with heme-proteins^35^. Similarly, scattering spectra of single gold nanorods embedded in HITC dye were shown to exhibit both monomer and H-aggregate absorption bands^36^. These results inspired us to quantitatively investigate the issue of strong coupling between molecular layers and well-characterized single plasmonic nanorods. Single nanoparticle measurements have a number of advantages compared to ensemble studies. In particular, the absence of inhomogeneous broadening allows for precise determination of decay rates. Here, we report that single silver nanorods can interact strongly with thin layers of surrounding TDBC dye molecular J-aggregates, resulting in highly pronounced exciton-induced transparency dips in both scattering and extinction spectra at room temperature. We discuss the critical role of particle volume, molecular resonance width and cavity-exciton detuning in determining the character of the hybridized spectra. Our observations indicate a transparency of about 50% and a ~ 100 meV vacuum Rabi splitting. To our knowledge, this is the largest exciton-induced transparency experimentally shown to date on the single isolated metal nanoparticle level. In dimer structures, where the field can be concentrated even further, Rabi splitting of ~ 400 meV has been reported very recently^37^. We also show that low power laser illumination completely quenches the transparency dip due to irreversible degradation of the J-aggregate into monomers. This highlights the importance of J-aggregate stability for possible nonlinear applications. Our results suggest routes towards further optimization of the hybrid plasmon-exciton systems and possible applications, including active plasmonic platforms for photonics, electro-optical applications, classical and quantum optical communication and logic schemes.

This paper is organized as follows. First, we focus on experimental observation of spectral dips in single-nanorod scattering and extinction spectra and discuss the physical origin of the dips in terms of the exciton-induced transparency and enhanced absorption. Then, we compare spectra of coupled and uncoupled systems by photobleaching J-aggregates. We numerically model the structures to show that the spectral dips are formed in both scattering and absorption spectra, proving that the system is in the exciton-induced transparency regime. Finally, we analyse the effect of nanoparticle volume on the depth and width of the spectral dips and argue that the most pronounced dips are formed in the case of the smallest rods.

## Results

### Experimental observation of plasmon-exciton transparency

**Figure 1:**
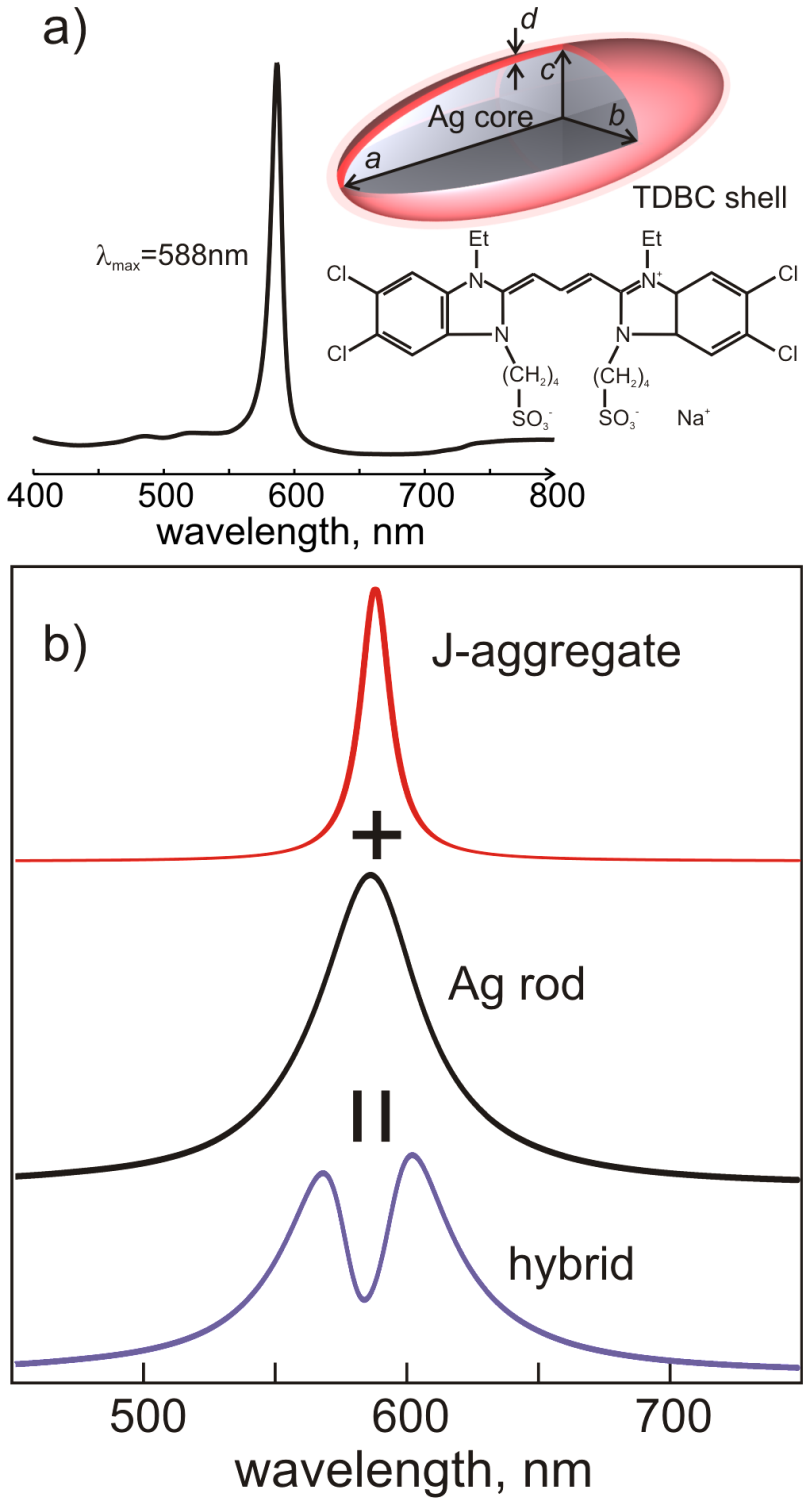
(a) A sketch showing the Ag core – molecular shell spheroid with semiaxes *a*, *b*, *c*, uniform shell thickness – *d* and the chemical structure of TDBC. Inset: the absorption spectrum of 10^−5^ M TDBC J-aggregate in aqueous solution containing 5 mM NaOH. (b) Absorption spectrum of free J-aggregate (red) and scattering spectrum of a bare Ag nanorod (black), which upon interaction, give rise to a hybrid plasmon-molecule spectrum (purple).

Figure 1a shows a sketch of the system under study and the chemical structure of the TDBC monomer (5,6-Dichloro-2-[[5,6-dichloro-1-ethyl-3-(4-sulfobutyl)-benzimidazol-2-ylidene]-propenyl]-1-ethyl-3-(4-sulfobutyl)-benzimidazolium hydroxide, inner salt, sodium salt). The structure consists of an elongated silver core surrounded by a thin molecular layer. In experiments, the structures are supported by a glass substrate. We choose to work with J-aggregates because of the high oscillator strength of their electronic excitations, which is a necessary condition for observation of exciton-induced transparency. The J-aggregated form of TDBC is particularly attractive because its absorption spectrum is very simple, that is, possessing only a single intense and narrow band at around 588 nm (see inset Fig. 1a and for example Ref. 38). There is a qualitative similarity between the absorption of J-aggregates and the narrow zero-phonon line (ZPL) typically emerging in spectra of organic chromophores at low temperatures^39^. An advantage of J-aggregates is that their absorption is intense and narrow even at room temperature. Figure 1b shows spectra of a free J-aggregate and a single silver nanorod, as well as the spectrum of a combined system, exhibiting a pronounced transparency dip as a result of strong interaction between excitons and plasmons. The spectra were calculated in the quasi-static approximation (see Supplementary Information) and demonstrate that a simple metal core/J-aggregate shell hybrids may exhibit a very peculiar spectroscopic response.

**Figure 2:**
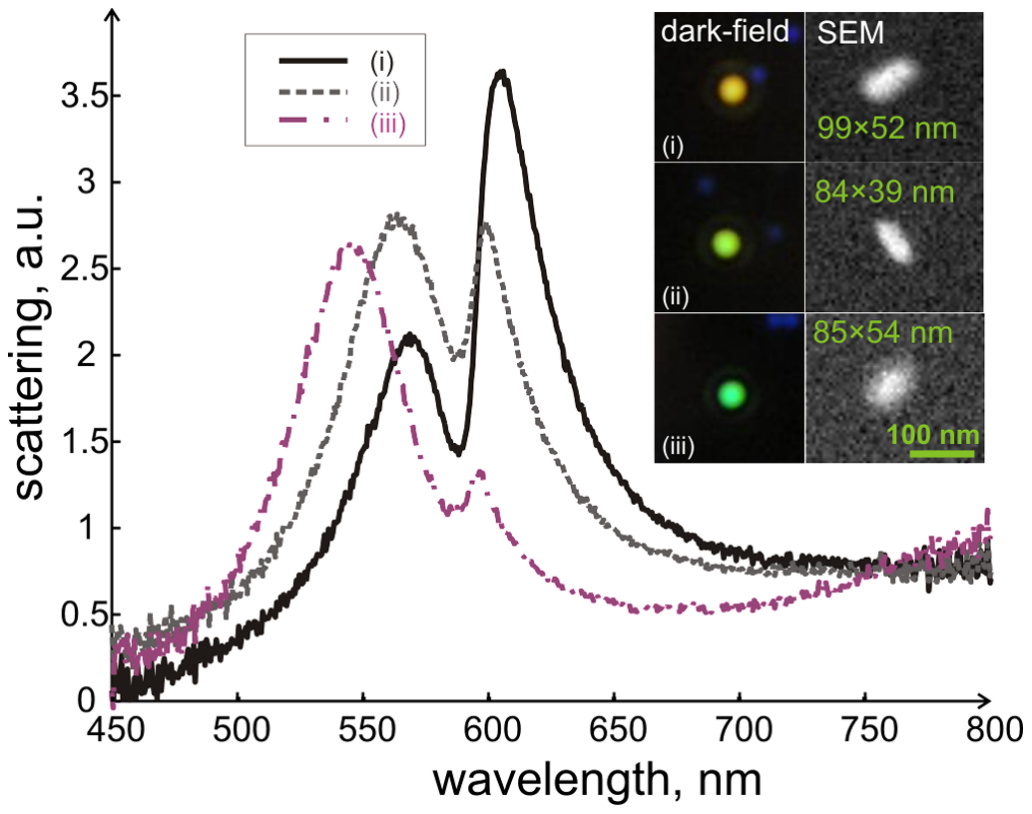
Experimental dark-field scattering spectra of three individual Ag nanorod particles surrounded by a TDBC J-aggregate shell. The inset shows dark-field and SEM images of the corresponding particle. The spectra were taken under unpolarized white light illumination. The scale bar is 100 nm.

Figure 2 shows experimental scattering spectra from three typical silver nanorods that have slightly different dimensions and therefore colours. Dark-field optical microscopy and SEM images of the nanorods are shown in the inset. It is well-known that silver and gold nanoparticles support collective oscillations of surface charges - localized surface plasmon resonance (LSPR). Moreover, for anisotropic particles, longitudinal and transverse resonances can be identified. The J-aggregate absorption line overlaps only with the longitudinal LSPR for the rods studied in this work. The experimental scattering spectra shown in Fig. 2 clearly exhibit not only longitudinal plasmon bands but also molecular absorption features, which appear as significant spectral dips at ~ 588 nm.

**Figure 3:**
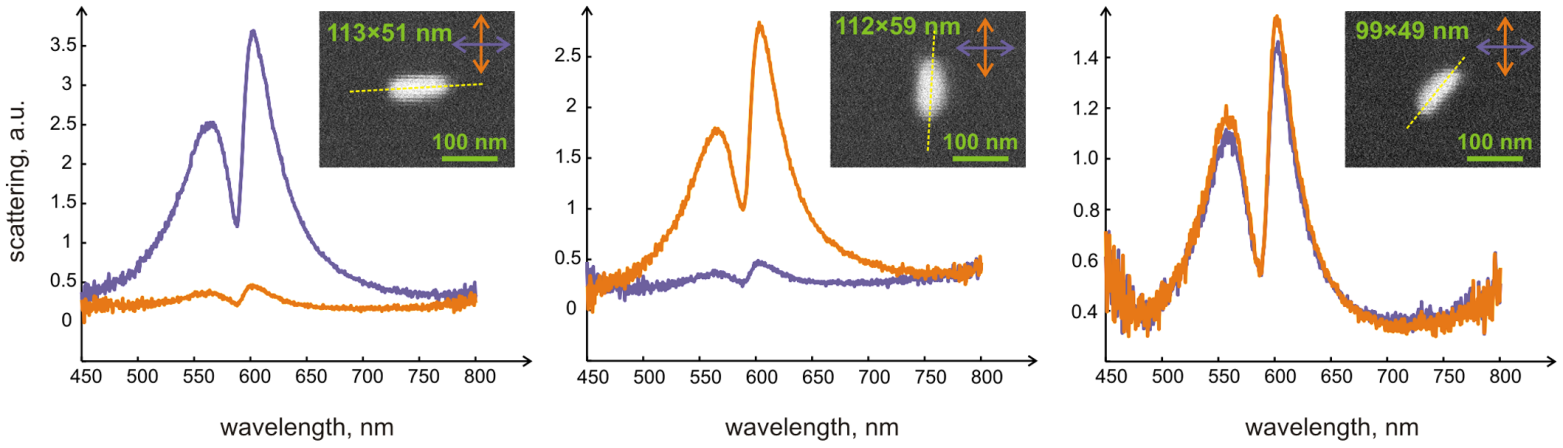
Scattering spectra of three silver nanorods excited with polarized white light. Purple and orange lines show horizontal and vertical polarization direction respectively. (a) Spectrum of nearly horizontally oriented rod. (b) Spectrum of nearly vertically oriented rod. (c) Spectrum of ~ 45° tilted rod. Insets show SEM images of the rods. In all cases longitudinal polarization dominates the scattering spectra.

To demonstrate that it is only the longitudinal LSPR that interacts with the J-aggregate and therefore gives rise to the observed behaviour, we performed a series of polarization-resolved measurements, as summarized in Figure 3. The results show that when the incident light is polarized along the major axis of the nanorods, the scattering intensity is maximized, while the signal is strongly suppressed when the polarization is perpendicular to that axis. SEM images of the studied nanorods are represented in the insets of Fig. 3 and show that orientations of silver nanorods are in good agreement with the polarization-resolved scattering data. The polarization-resolved measurements indicate that, within the size range of silver nanorods studied here, the transverse LSPR appears in the deep blue or even near-UV spectral region (<400 nm), which is beyond the spectral range of our experimental setup (also see SI). One can, therefore, safely assume that it is only the longitudinal LSPR that contributes to the interaction with the J-aggregate. For that reason, unpolarized incident light was used in further experiments in order to efficiently excite all silver nanorods irrespectively of their random in-plane orientation.

**Figure 4:**
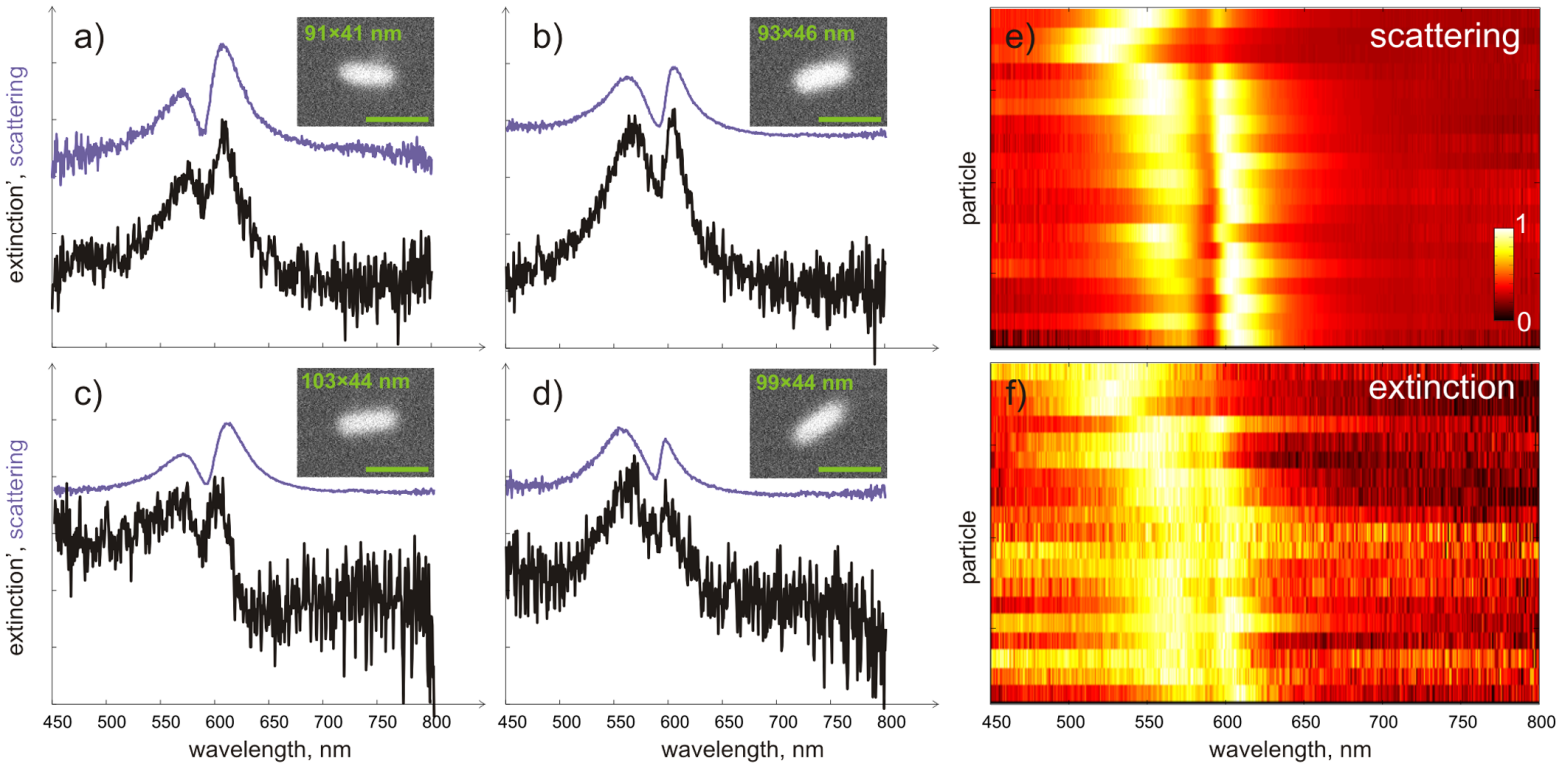
(a–d) Single-nanoparticle scattering *and* extinction spectra. Insets show SEM images of the rods (scale bar 100 nm). Note that the scattering contribution to the extinction is reduced to about ~ 1/3 of its original value, due to high NA of the collecting optics (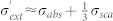). Note spectral dips in both scattering and extinction data. (e–f) colour-coded normalized scattering and extinction spectra for 20 different silver nanorods.

One may argue that the spectral dips in the scattering spectra of the individual rods shown in Figs. 2–3 arise not due to strong plasmon-exciton coupling or exciton-induced transparency but simply due to enhanced absorption in the dye layer. While both phenomena can lead to observation of spectral dips in scattering spectra, it is disputable which mechanism dominates under particular circumstances^40^. Although several previous works argue for strong coupling, clear evidence for strong coupling, that is observation of spectral dips in single-particle absorption, have not yet been reported^34^,^35^,^36^,^37^. Typically either scattering or ensemble-averaged extinction measurements are presented. The latter may still be dominated by scattering for large particles. To address this issue, we performed a series of *single-nanoparticle* *extinction* measurements. If enhanced absorption is the dominant process, one would expect no spectral dip but rather an additional spectral peak at the J-aggregate line in the absorption spectrum of the hybrid system (see SI, Fig. S2). To the contrary, if the molecule-particle system is in the strong coupling regime, dips should appear both in absorption and scattering. We measure extinction by illuminating a single nanoparticle from the air side and collecting transmitted light with 100 × NA = 1.3 oil immersion objective. The measurements are referenced by transmission through the bare air-glass interface. The single-nanoparticle extinction measurements together with the scattering results are shown in Figure 4. Spectral dips at the position of J-aggregate absorption are clearly visible in both scattering and extinction spectra for all investigated nanorods, suggesting they are indeed due to exciton-induced transparency. Moreover, as demonstrated in detail in the SI, the contributions of absorption and scattering to the measured extinction become comparable and about 1/3 and 2/3 correspondingly, as a result of high numerical aperture of the collecting objective. Usage of high NA optics is thus advantageous in experiments where the absorption contribution is required to be maximized, as is the case here. These results provide further evidence of strong coupling in the investigated plasmon-molecule system.

### Photodegradation of J-aggregate

Although single-nanoparticle extinction measurements presented above provide additional evidence for the strong-coupling nature of the observed spectral dips, our measurement setup does not allow to collect pure absorption cross-section of a single nanoparticle and, thus, to unambiguously discard the enhanced absorption possibility. Experiments presented in this section aim at gaining further knowledge about the system by comparing experimental results to numerical simulations that allow for explicit evaluation of absorption cross-sections. On top of that, these experiments also provide useful information about fluorescence of J-aggregates on silver nanorods.

**Figure 5:**
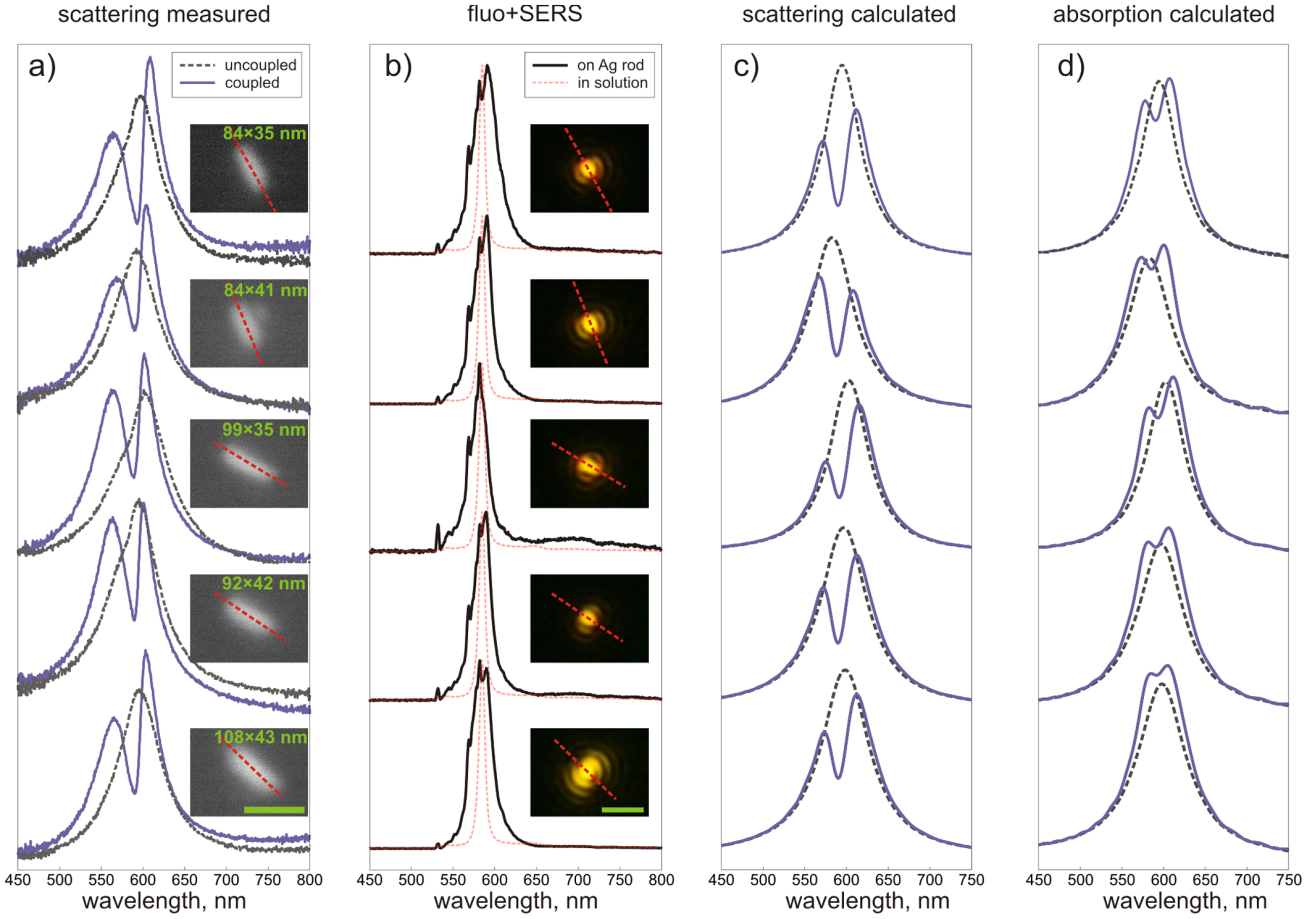
(a) Scattering spectra of a single Ag nanorods before and after illumination with a 532 nm laser. Inset shows SEM images of the corresponding nanorods. Scale bar is 100 nm. (b) Fluorescence spectra of TDBC on silver nanorod (solid) and free J-aggregates in water solution (dashed). Fluorescence spectra on Ag rods are accompanied by sharp SERS lines. Inset shows fluorescence images of the nanorods. Scale bar is 1 μm. Note that fluorescence images are symmetric with respect to the nanorods orientation. (c–d) FDTD simulations of scattering (c) and absorption (d) cross-sections of bare (dashed) and covered (full) Ag nanorods. Notice spectral dips in both scattering and absorption.

A continuous-wave 532 nm laser under relatively low irradiance (1 kW/cm^2^) was used throughout this study. Interestingly, we found almost a complete disappearance of transparency dips even under this moderate irradiance, enabling a direct comparison between the coupled and uncoupled plasmon-exciton systems. Considerable photodegradation of J-aggregate occurred on the timescale of only several seconds. Figure 5a shows a comparison between the particles' spectra before and after laser illumination. SEM images of the corresponding nanorods are shown in the inset. After photobleaching a single well-defined peak at the position of longitudinal LSPR is observed for all investigated nanorods. In the case of coupled particles, the spectra look similar to those previously shown (see Figs. 2, 3, 4). The coupled particle spectra are broadened in comparison to the uncoupled ones, thus indirectly signaling strong interaction between excitons and plasmons. To get a further insight into the problem, we have modeled our nanoparticles using a commercial FDTD package (Lumerical). A detailed description of the calculation procedure is given in the Methods section. Figures 5c and 5d show calculated scattering and absorption cross-sections for both coupled and uncoupled cases. Note that spectral dips at the J-aggregate absorption line appear in both scattering *and* absorption indicative of a true transparency regime. The agreement between the simulated and experimental data is rather good in terms of resonance positions, splitting widths and depths. There is, however, an interesting discrepancy between the experimental and simulated data in that the integrated scattering cross-section for both coupled and uncoupled cases are very similar in experiments, while in simulations bare rods have always greater scattering cross-sections. We speculate that the origin of this phenomenon is that the photoproduct of the dye might be highly absorbing in a broad spectral range, thus, effectively lowering the uncoupled particle spectra.

Fluorescence spectra collected during the first 25 s of exposure are shown in Fig. 5b and are compared to the fluorescence of free J-aggregates in water solution. Previous works^16^,^41^ argue that strong coupling between a J-aggregate and a plasmonic cavity should cause splitting of a fluorescence signal into upper and lower polariton branches by analogy to elastic scattering and extinction spectra. The upper polariton, however, was reported not to fluoresce at room temperature, resulting in a red-shifted fluorescence that originates exclusively from the lower polariton branch^16^,^41^. As is seen in Fig. 5b, the fluorescence from J-aggregate on Ag rods is considerably broader than that of a free dye, however, no red shift or splitting was detected. This is likely due to photo-degradation of the J-aggregate that occurs faster than the fluorescence collection time. Fluorescence microscopy images of the corresponding nanorods are shown in the inset of Fig. 5b. The images are symmetric with respect to the dashed red lines, which show the orientation of the nanorod as deduced from the SEM, suggesting the emission profile is dipolar and oriented along the major axis of the nanorods^42^,^43^. This implies that the fluorescence emission occurs via the longitudinal plasmon mode and hence the J-aggregates are very close to the metal surface. In addition, fluorescence spectra are accompanied by sharp peaks at 569.2, 573.6, 578.7 and 582.0 nm (corresponding to 1228, 1363, 1517 and 1615 cm^−1^) which are likely due to surface-enhanced Raman scattering (SERS) of TDBC. The SERS spectrum shows the vibrational bands that are in reasonable agreement with earlier works^44^, considering ~ 15 cm^−1^/pixel spectral resolution of the 150 g/mm grating used.

Irradiance of 1 kW/cm^2^, in principle, should not induce any significant temperature rise in and around the particles under steady-state conditions (estimated to be ≈ 1 K from 
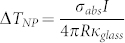
 and assuming *R* = 40 nm silver sphere for the absorption cross-section)^45^,^46^. Our results imply that the J-aggregate is nevertheless decomposed, probably due to insufficient heat dissipation into the substrate and enhanced absorption in the dye layer, indicating the importance of stability issues for J-aggregates in such combined systems. Stability may be potentially achieved, for example, by cooling the system to cryogenic temperatures and performing experiments under vacuum^19^ or by embedding the whole system into a silica shell^29^.

In summary, the results presented in this section demonstrate spectral dips in both scattering and absorption cross-sections – a direct indication of a true transparency regime. This was concluded by comparing the coupled and uncoupled plasmon-molecule systems and numerical simulations.

### Effect of nanorod volume on the coupling strength

As is demonstrated in previous sections, nanoparticle-molecule system can indeed be found in a transparency regime. In this section, we specifically focus on parameters affecting the coupling strength, especially concentrating on the nanoparticle volume – a parameter that determines the plasmon radiative decay. We start by considering an intuitive coupled-oscillator model, where both plasmons in the core and excitons in the shell of our structures are modeled as two classical dissipative harmonic oscillators, coupled to each other with a rate *g*^47^. Following previous works^2^,^47^, we write the scattering cross-section of the combined system as: 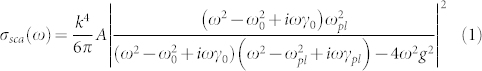
where *A* is a scattering amplitude, proportional to the particle volume squared (~*V*^2^), and *ω_pl_* and *ω*_0_ refer to the LSPR and molecular resonance frequencies, respectively. When 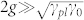 or, alternatively, 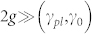 the system enters the strong coupling regime, meaning that the coupling overwhelms any decoherence processes. Clearly, to approach the strong coupling regime, the coupling strength *g* has to be increased, while the decoherence channels *γ_pl_* and *γ*_0_ are suppressed. In case of plasmonic nanoparticles, the requirement for strong coupling can be simplified, as typically 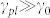, and so it is enough to satisfy 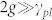.

One single parameter that affects both *g* and *γ_pl_* in our experiments is the nanorod volume. The effect of the particle volume on the plasmon decay is rather straightforward, since its radiative contribution scales as 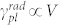. On the other hand, the coupling strength scales as 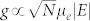, where *N* is the number of molecules, *μ_e_* is the molecular transition dipole moment and 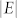 is the electric field at the position of the molecule. The particle volume enters *g* through 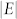, which scales as 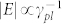. In addition, the number of molecules *N* is approximately proportional to the nanoparticle surface area *S*, and so the coupling strength should scale as 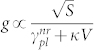, where 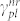 is the Ohmic loss and 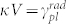. We thus expect that *γ_pl_* should decrease, while *g* slightly increase with reduction in the nanorod volume, thus pulling the systems towards the strong coupling regime. These expectations are confirmed by calculations (see Fig. S5).

**Figure 6:**
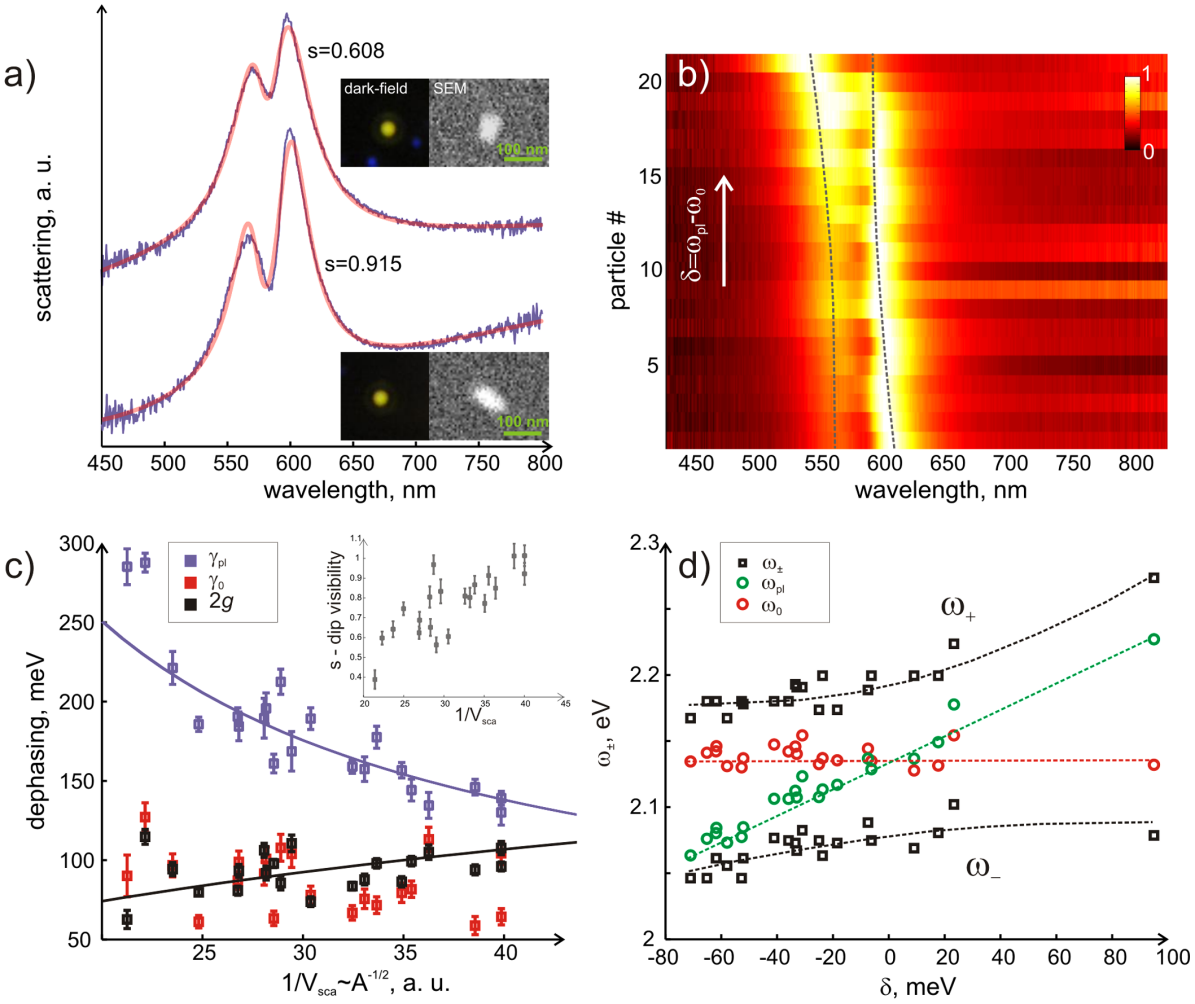
Scattering spectra and SEM images of 21 different Ag nanorods fitted to a coupled-oscillator model. (a) Spectra and fits of two representative rods of high and low coupling strength (spectra are displaced for clarity). The inset shows dark-field images and SEM images. (b) Normalized scattering spectra for 21 individual nanorods arranged in the order of increasing detuning. The dashed lines follow the position of the hybrid resonances. (c) Dephasing rates extracted from data in b) by fitting to the coupled-oscillator model and shown as a function of 1/*V_sca_*. The inset shows a dimensionless depth of the dip parameter 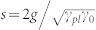 as a function of inverted volume. Solid curves are the fits of *γ_pl_* and 2*g* that cross at about 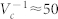 – the onset of strong coupling regime. (d) Molecular (*ω*_0_), plasmon (*ω_pl_*) and hybrid (*ω*_+_ and *ω*_−_) resonances shown as a function of detuning. The dashed lines are guides for the eye.

To verify this experimentally, we collected scattering data from ~ 20 different individual silver nanorods of slightly different volumes. Both optical measurements and SEM data were collected for all of these particles and electron microscopy confirmed that in all cases the particles were single isolated silver nanorods. We further used Eq (1) with an additional term characterising the background to fit the experimental data. The results are shown in Figure 6. In panel a) two representative spectra and their fits are shown. The coupled oscillator model seems to adequately represent our experimental data. The model contains several parameters, which we are now at a position to analyze in depth. First of all we extract the difference between the plasmon *ω_pl_* and molecular resonances *ω*_0_ - the detuning *δ*, and arrange the spectra in the order of increasing *δ*. Normalized scattering intensities arranged in this way are shown in Fig. 6b. Note that the mode anti-crossing behaviour is visible, however, probably due to heterogeneity of the nanoparticles, this behaviour is less apparent than the theoretical analysis would suggest (see SI, Fig. S5). We further extract the plasmon *γ_pl_* and molecular resonance *γ*_0_ damping as well as the coupling rate *g*, and plot them as a function of the inverted nanoparticle volume (Fig. 6c). The particle volume is extracted directly from the scattering measurements as *V_sca_* ∝ *A*^1/2^. This ‘optical’ volume is proportional to the square root of the parameter *A*, which is also obtained through fitting the data. The optical volume correlates well with the values deduced from SEM (see SI, Fig. S4), although electron microscopy images are 2D and thus assumptions about the unknown rod height have to be made in order to estimate the real geometrical volumes. To exclude these additional assumptions from consideration, we will use the optical volume in the discussion from here on.

As expected, plasmon damping significantly decreases as the volume becomes smaller, due to the decreased radiative contribution (Fig. 6c). The molecular dephasing *γ*_0_ and the Rabi splitting 2*g*, both do not change much, with only slight increase in 2*g* seen with the reduction in volume. Fluctuations of 2*g* and *γ*_0_ as a function of *V*^−1^ are significantly above the error bars, suggesting they are a reflection of realistic particle and molecular coverage heterogeneity presented in the experiments.

The coupling strength in our experiments never exceeds the plasmon dephasing rate and the inequality *γ_pl_* > 2*g* ≥ *γ*_0_ always holds, at least approximately. We therefore never reach the strong coupling regime. However, for the smallest particle volumes the system is close to its onset, as 2*g* approaches the plasmon width at about 110 meV. To demonstrate this further, we plot the depth of the transparency dip (defined as dimensionless parameter: 
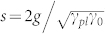
) versus the inverse volume *V*^−1^ in the inset of Fig. 6c. It is clear that the dips are more visible upon reduction of the particle volume. Moreover, a clear trend of decreasing the plasmon width as a function of *V*^−1^ suggests that if the data is extrapolated to even smaller nanorod volumes, the coupling strength will become greater than the plasmon dissipation at some point, and thus the system will enter the strong coupling regime. This is shown in Fig. 6c by fitting *γ_pl_* and 2*g* with the following approximate equations: 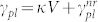 and 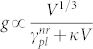 (*κ* = 4500 and 
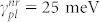
). The crossing of the *γ_pl_* and 2*g* fits occurs at *V*^−1^ ≈ 50, i.e. for silver rod volumes that are only 1.25 times smaller than the smallest rods in this study. This is further demonstrated in more detail in the SI.

We finally extract the plasmon and molecular resonance positions, as well as positions of the upper and lower polariton branches (denoted as *ω*_+_ and *ω*_−_ respectively) and plot them as a function of detuning. The result is shown in Fig. 6d. The anti-crossing splitting of about 100 meV is seen, although again less apparent than in simulations (see SI, Fig. S5). The reason for this, as was already mentioned, can be the heterogeneity in number of molecules surrounding each individual nanoparticle and the heterogeneity of nanorods themselves.

Overall, we show that particle volume plays an important role in determining the coupling strength in combined J-aggregate/plasmonic cavity system via plasmon radiative damping. It thus can be anticipated that particles with extreme values of surface-to-volume ratios, such as for example nanoplates and nanoprisms would show even stronger coupling under similar conditions^48^.

## Discussion

In conclusion, we have observed very prominent transparency dips in scattering, extinction and absorption spectra of individual silver nanorods covered with thin layers of TDBC J-aggregates. We have shown that plasmon damping, especially via the radiative channel, plays an important role in the formation of efficient hybrid systems. Our findings evidently indicate that the coupling strength increases for smaller particle volumes due to efficient suppression of plasmon radiative damping. Ultimately, the results also imply that if the particle volume is further reduced, in principle, even stronger coupling scenarios are possible. The simulations and experimental data suggest that the strong coupling limit would be reached for nanorods of about 50–60 nm in length, assuming the molecular coverage is similar to what is presented in this work. In contrast to previous studies, we report single-nanoparticle extinction spectra where the transparency dips are also present, which allows us to conclude more confidently that the system is indeed in the exciton-induced transparency regime. Further evidence of strong interaction between plasmons and excitons is gained upon comparison between the experimental and simulated data.

## Methods

### Sample preparation

The molecule-particle structures were prepared by incubating an as-received water solution of citrate-stabilized Ag nanoparticles (BBI International) with 20 μM TDBC J-aggregates (FEW Chemicals) for 24 hours, followed by twice repeated centrifugation and dissolution step to remove excess dye^27^. The particles were then immobilized on a glass substrate pre-coated with poly-lysine (0.25 mg/mL) and containing gold marker grids (produced by electron beam lithography), washed with excess of MilliQ water and dried under a stream of nitrogen. Note that silver nanorods used in this study were citrate stabilized, implying that molecular aggregate binging probability should not be very different between the rods' sides and ends, as opposed to the CTAB stabilized nanorods where preferential binding occurs at the nanorods ends. In addition, we have also prepared samples by incubating nanorods with higher concentration of TDBC (0.1 mM), however the scattering data (not shown) was very similar to the case of 20 μM, implying that the metal surface is completely saturated with the J-aggregates even at 20 μM concentration.

### Optical measurements

For single-particle measurements the samples were analysed in an inverted optical microscope (Nikon TE2000) in dark-field (collection objective: 100 × NA = 0.5 Nikon) and bright-field (collection objective: 100 × NA = 1.3 Nikon) for scattering and extinction measurements, respectively. The tungsten halogen filament of the microscope (100 W) was used as an incident light in all elastic scattering experiments. The dark-field condenser (air, NA = 0.85–0.9 Nikon) was used for both extinction and scattering measurements. A linear polarizer was installed in front of the condenser in the case of polarization-resolved measurements. Scattering and extinction were collected by a multimode fibre and sent to a spectrometer (Shamrock, Andor) equipped with a thermoelectrically cooled CCD detector (Andor). 50 μm and 200 μm core fibres were used for extinction and scattering measurements, respectively. In experiments shown in Fig. 4, a 50 μm fibre was used to collect both extinction and scattering data of the same nanoparticle. For fluorescence measurements, linearly polarized 532 nm continuous-wave laser (irradiance ~ 1 kW/cm^2^ at the sample) was used as an excitation. A set of excitation, dichroic and long-pass emission filters (Chroma) was used to remove the laser light. All optical experiments were performed under a stream of nitrogen to reduce photo-degradation of both J-aggregates and silver nanoparticles.

### Electron microscopy

Low-vacuum (0.2 mbar) scanning electron microscopy (SEM) under 5 keV acceleration voltage was used for detailed structural characterization of Ag nanoparticles. The low-vacuum SEM allows for facile observation of samples on non-conductive substrates, such as glass. No J-aggregates could be detected in the SEM measurements.

### FDTD simulations

A commercial FDTD package (Lumerical) was used to simulate both covered and uncovered nanorods residing on the glass substrate. As an input, the model uses geometrical parameters of the rods deduced from the SEM. The rods were assumed to have equal height and width (*a* > *b* = *c*) and to be located on an air-glass interface. The refractive index of glass was allowed to vary slightly to get a match between experimental and simulated resonances. This is justified by the fact that in experiments the rod's heights are not precisely known. The dispersion of molecular layer was assumed to be Lorentzian: 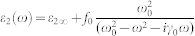. The J-aggregate absorption line was set to 595 nm (*ω*_0_ = 2.08 eV), while the width of the resonance was set to *γ*_0_ = 50 meV. The dimensionless oscillator strength was set to *f*_0_ = 0.05 and the width of molecular layer was always kept at 2 nm. Parameter *ε*_2∞_ was assumed to be 1.45^2^, that is a typical value for dense molecular layers. In case of an uncoupled nanoparticle *f*_0_ was set to 0. The choice of the oscillator strength and the molecular shell thickness in the simulations is justified by the reasonable agreement with the scattering and extinction experiments and the absorption of the free dye presented in Fig. 1.

## Author Contributions

G.Z. and T.S. designed and conducted experiments, T.J.A. performed numerical simulations, G.Z., G.J., P.J., T.J.A., M.K. and T.S. analyzed the data, all authors reviewed the manuscript.

## Supplementary Material

Supplementary Information Supplementary info
