# Supplementary material for: Approaching the strong coupling limit in single plasmonic nanorods interacting with J-aggregates
Source: Sci Rep. 2013 Oct 29;3:3074. doi: 10.1038/srep03074 (PMC3810662; doi:10.1038/srep03074)
Supplement: Supplementary Information — Supplementary info [file srep03074-s1.pdf]

Supplementary information for

“Approaching the strong coupling limit in single plasmonic nanorods interacting with J-aggregates.”

by Gülis Zengin, Göran Johansson, Peter Johansson, Tomasz J. Antosiewicz, Mikael Käll, and Timur Shegai.

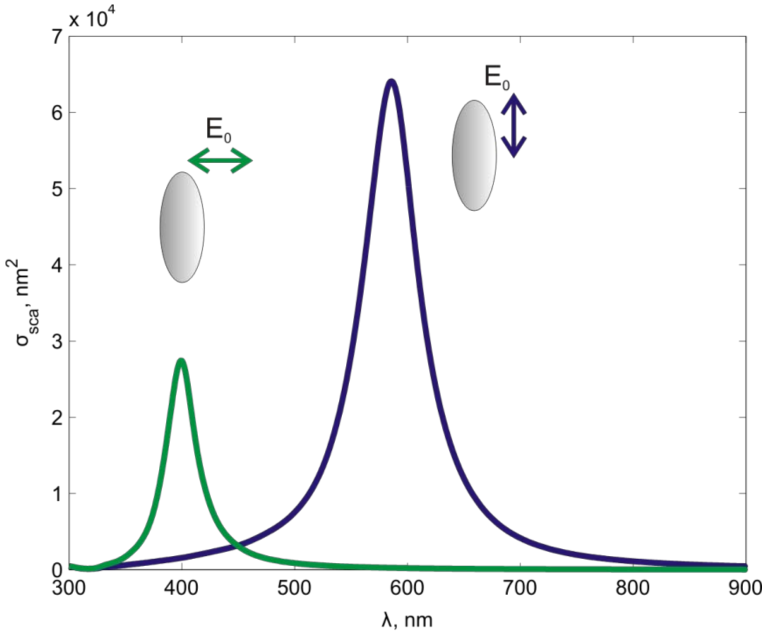

Figure S1. Polarization-resolved scattering of a silver ellipsoid with dimensions of 100×40×40 nm embedded into homogeneous optical medium with n_{eff}=1.35. Longitudinal LSPR appears around 590 nm, while transverse LSPR around 400 nm. Thus it is only the longitudinal LSPR that spectrally overlaps with the J-aggregate absorption line (~588 nm).

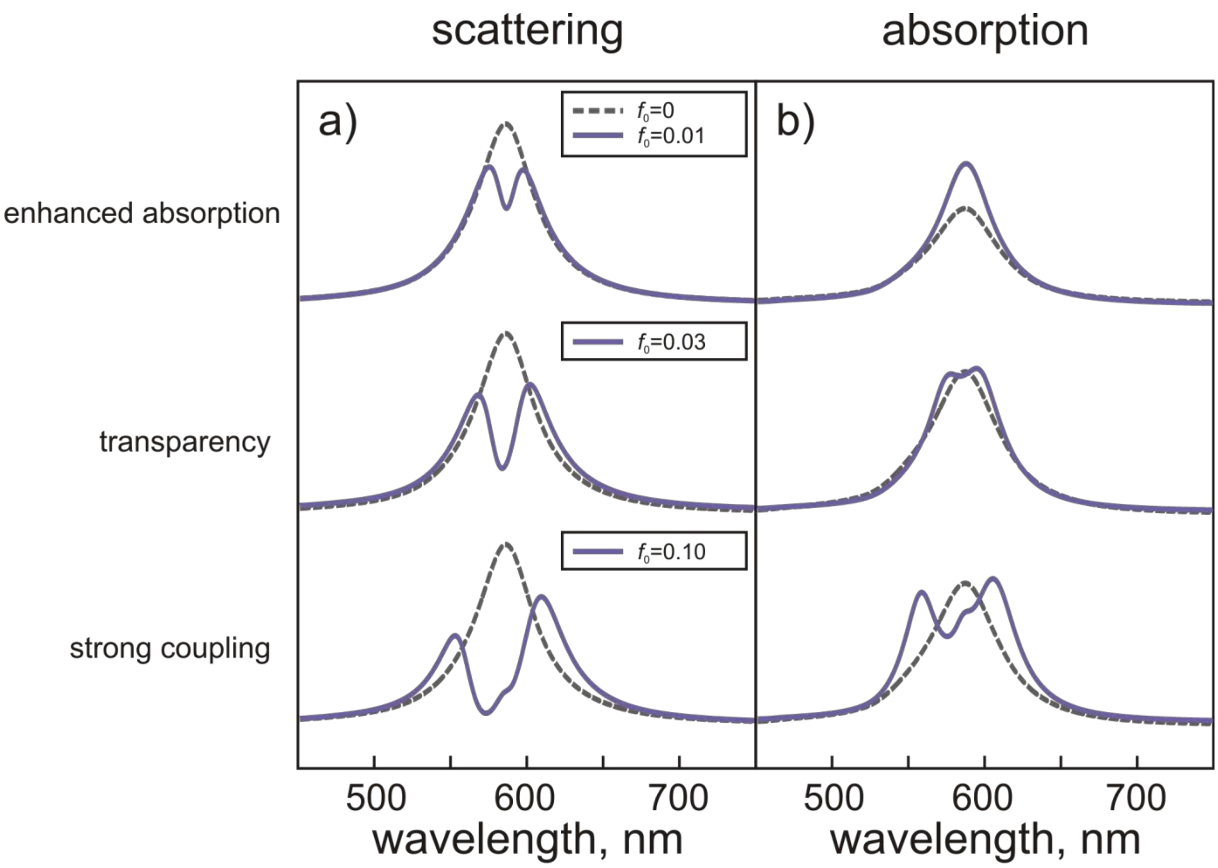

Figure S2. Scattering and absorption as calculated by the quasi-static model for silver ellipsoid with dimensions of 90×35×35 nm covered with 2 nm J-aggregate layer with oscillator strength of 0.01, 0.03 and 0.1 respectively. Molecular resonance: 588 nm, width 50 meV. Dashed lines show spectra of uncovered ellipsoids calculated for f_0=0. All particles were embedded into homogeneous optical medium with n_{eff}=1.36. Notice splitting in both absorption and scattering cross-sections at f_0=0.03 and f_0=0.1. No splitting in absorption is visible for f_0=0.01, but a dip in scattering is still present. Consult SI text for further details.

Single-nanoparticle extinction measurements: Attenuation of transmission in an extinction measurement occurs due to absorption in and scattering by the nanoparticle. It is known that nanoparticles residing on strongly mismatched interfaces, such as of air and glass, preferentially scatter to the optically denser medium, i.e. the glass [1-3]. Because we measure the transmission with a high NA objective from the glass side, we also collect a considerable part of scattering, therefore effectively reducing its contribution to extinction. For these reasons we will refer the measured extinction as σ'_{ext} and not σ_{ext}. The σ'_{ext} is composed of the nanoparticle's absorption and scattering that is *not* collected by the microscope's objective. Integrating the radiation pattern from a single nanorod over the solid angle corresponding to NA=1.3 oil-immersion objective shows that the fraction of *not* collected scattering is only about 1/3 (see Fig. S3). Therefore the measured extinction cross-section can be written as: $\sigma'_{ext} \approx \sigma_{abs} + \frac{1}{3}\sigma_{sca}$. Furthermore, for particles with dimensions of 100×50 nm, which are the typical dimensions of our nanorods, the ratio between absorption and scattering at the LSPR is about $\frac{\sigma_{abs}}{\sigma_{sca}} \approx 0.15$. Thus, the relative contributions of absorption and scattering to the measured extinction is about ~1/3 for the absorption and ~2/3 for the scattering respectively. Consequently the measured single-particle extinction, σ'_{ext}, is still dominated by scattering, but the contributions are nevertheless comparable. One could further increase the absorption contribution by either increasing the NA of the collecting objective and/or by collecting the light back-scattered to the air side with additional collecting optics. For particles smaller than 100×50 nm used for estimations here, the absorption contribution will further increase.

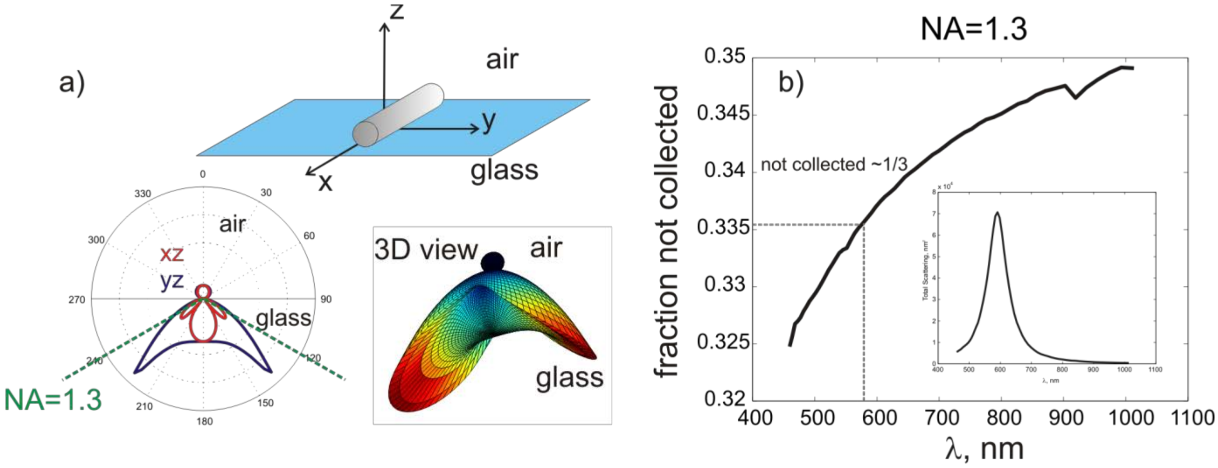

Figure S3. a) Angular distribution of scattering for a 100×50×50 nm silver nanorod residing on air-glass interface as calculated by Green's function method [4]. b) Fraction of scattering *not* collected by NA=1.3 oil immersion objective as a function of wavelength. Inset: scattering spectrum of silver nanorod.

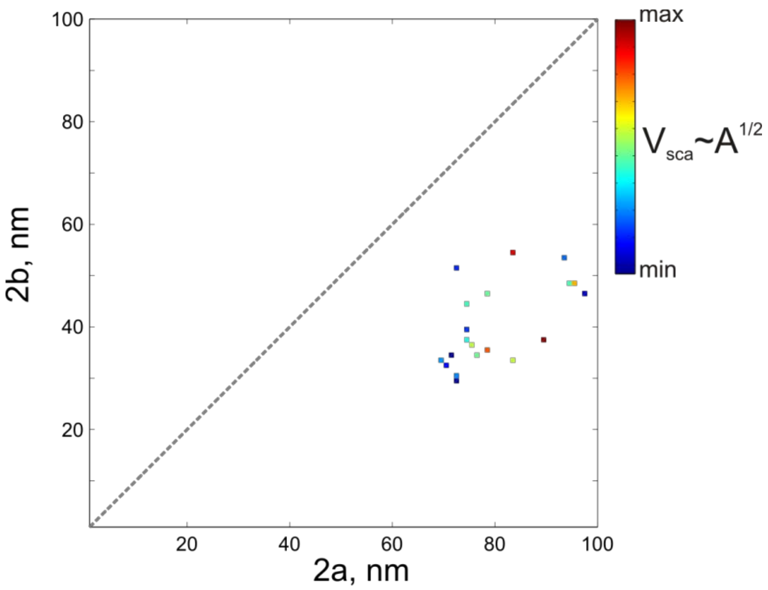

Figure S4. Particle dimensions as deduced from SEM plotted versus color-coded scattering volume obtained from the coupled oscillator fits. Plot shows good overall correlation between SEM data and scattering fits, with some deviations, which are probably due to unknown rods' heights.

Analytical model: In this section, we present an analytical quasi-static model that under realistic parameter values, reproduce our experimental findings. We use this model to verify that the particle volume is a critical parameter that affects the coupling strength. The model was used to calculate scattering and absorption spectra shown Figs. S1-S2, S5 and to qualitatively verify the effect of nanoparticle surface-to-volume ratio to be able to compare to the results shown in Fig. 6 of the main text.

We start by assigning dispersive permittivities $\varepsilon_1(\omega)$, $\varepsilon_2(\omega)$ and ε_3 (wavelength-independent) to the corresponding layers of the core-shell structure (see Fig. 1a main text). For simplicity we assume here all permittivities to be isotropic, although the transition dipole orientation can certainly play a role [5]. Such a system can be described in a quasi-static approximation if the semiaxes are much smaller than the wavelength (a, b, $c \ll \lambda$). We also introduce a so-called modified long-wavelength approximation (MLWA) which effectively takes into account radiative damping and dynamic depolarization thus producing realistic spectral width and resonance position [6, 7]. The polarizability of such a core-shell structure is given by [8, 9]:

$$\alpha_{0,i=x,y,z} = \frac{4}{3}\pi a_2 b_2 c_2 \frac{(\varepsilon_2-\varepsilon_3)\varepsilon_a + f\varepsilon_2(\varepsilon_1-\varepsilon_2)}{(\varepsilon_3+L_{i,2}(\varepsilon_2-\varepsilon_3))\varepsilon_a + fL_{i,2}\varepsilon_2(\varepsilon_1-\varepsilon_2)} \tag{S1}$$

where $\varepsilon_a = \varepsilon_2 + (\varepsilon_1 - \varepsilon_2)(L_{i,1} - fL_{i,2})$, $f = a_1b_1c_1/a_2b_2c_2$ – i.e. the volume fraction of the core, $s_{1(2)} = a_{1(2)}$, $b_{1(2)}$, $c_{1(2)}$, - the inner and outer spheroid semiaxes and $L_{(x,y,z),1(2)}$ – the inner and outer geometrical factors [8]. The MLWA-corrected polarizability ($\alpha_{i=x,y,z}$) is obtained from α_0 by multiplying it by $\chi = (1-ik^3\alpha_0/6\pi-k^2\alpha_0/(4\pi s_2))^{-1}$ [6, 7], where $k = \sqrt{\varepsilon_3}\,\omega/c$ is the wave-vector in the surrounding dielectric.

Equation (S1) reduces to the expression for uncovered spheroids, i.e. $V\frac{\varepsilon_1-\varepsilon_3}{\varepsilon_3+L_i(\varepsilon_1-\varepsilon_3)}$, at both $f = 1$ and/or $\varepsilon_2 = \varepsilon_3$. The case of a covered sphere is also easily obtained by setting

$L_1 = L_2 = 1/3$. The permittivity of the metallic core is taken from experimental values of Johnson and Christy [10] and the permittivity of the dye was calculated according to:

$$\varepsilon_2(\omega) = \varepsilon_{2\infty} + 4\pi N_V f_{tr} \alpha_{mol}(\omega), \tag{S2a}$$

$$\alpha_{mol} = V_{mol} \frac{\omega_0^2}{(\omega_0^2 - \omega^2 - i\gamma_0 \omega)} \tag{S2b}$$

which assumes a single Lorentzian resonance behavior with physical meaning of parameters to be dimensionless oscillator strength (f_{tr}) and the number of dye molecules per unit volume (N_V). The molecular polarizability is directly related to the extinction coefficient which can be evaluated experimentally. We further introduce another dimensionless quantity that takes into account molecular concentration and volume, $f_0 = 4\pi N_V f_{tr} V_{mol}$, thus the permittivity of the dye layer is given by:

$$\varepsilon_2(\omega) = \varepsilon_{2\infty} + f_0 \frac{\omega_0^2}{(\omega_0^2 - \omega^2 - i\gamma_0 \omega)} \tag{S3}$$

The scattering, extinction and absorption cross-sections are further evaluated from (S1-S3) as $\sigma_{sca} = \frac{k^4}{6\pi} |\alpha_x|^2$, $\sigma_{ext} = k\mathrm{Im}(\alpha_x)$ and $\sigma_{abs} = \sigma_{ext} - \sigma_{sca}$ correspondingly. The molecular extinction coefficient was set to 10^5 $M^{-1}cm^{-1}$ (corresponding to the peak absorption cross-section of $\sim\sigma_{abs} = 3.8\times10^{-16}$ cm^2) and the molecular resonance was assumed at 588 nm in all cases. The concentration of molecules was taken to be in the interval of 10^{19-20} cm^{-3} and the width of the shell layer to be uniform and equal to 2 nm. These parameters seem to describe our experimental structures consisting of an Ag nanorod core and a TDBC J-aggregate shell rather well.

We further verified the effect of the nanoparticle volume on the coupling strength and qualitatively compared it to the experimental observations. The particle size and aspect ratio were systematically varied, while the plasmon resonance frequency position was preserved to

match the J-aggregate electronic transition. The results were calculated using Eq. (S1) and are given in Figure S5. The width of the molecular shell layer was always kept at 2 nm. The width of the molecular resonance was 10 meV and the concentration of molecules was taken to be 10^{20} cm^{-3}.

Inspection of Figure S5 shows the clear trend of an increasing transparency dip for higher surface-to-volume ratios. This is especially evident for particles with *S*/*V* greater than ~0.15 nm^{-1} where almost a complete invisibility of the hybrid system at the molecular resonance frequency is achieved. The radiative damping of the plasmon resonance is clearly reduced with the reduction of the particle volume, which in turn also leads to stronger coupling between excitons and plasmons. The surface-to-volume ratio thus has a double effect on the coupling strength: (*i*) it decreases the width of the plasmon resonance due to suppression of its radiative contribution and (*ii*) it increases the number of dye molecules per metal atom in the core. The result of this doubled process is shown in Fig. S5a-b.

To characterize the coupling strength quantitatively, we also fit the results obtained from the core-shell model to the coupled-oscillator model introduced in the main text. By doing so we extract plasmon and exciton dephasing rates γ_{pl} and γ_0, as well as the Rabi splitting $2g$. These parameters are shown in Fig. S5b. As is seen, the plasmon dephasing rate is significantly suppressed, while the coupling rate is slightly increased with increasing surface-to-volume ratio. Both of these effects, but especially suppression of radiative damping, lead to more and more apparent transparency, in agreement with the experimental findings. Saturation in g as a function of *S*/*V* is caused by entering the Ohmic-limited plasmon damping regime. In most of the cases a situation $\gamma_{pl} > 2g > \gamma_0$ is found, however, at the two highest values of *S*/*V*, $2g$ is faster than both γ_{pl} and γ_0 meaning that the system enters the strong coupling regime.

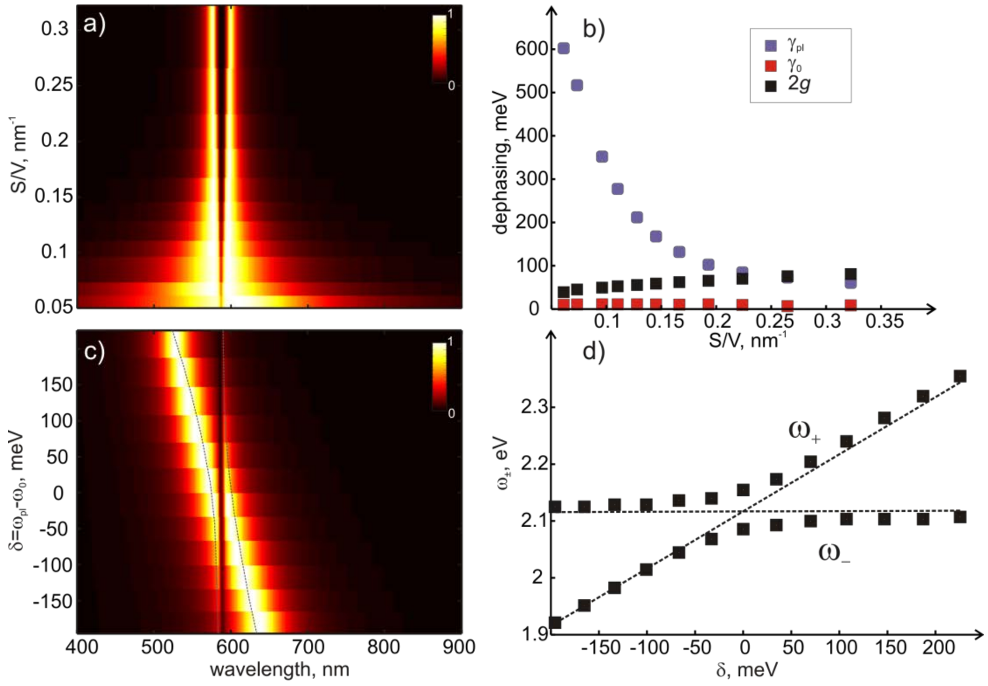

Figure S5. The effect of particle surface-to-volume ratio and plasmon-exciton detuning on coupling strength calculated using the core-shell model. a) The normalized scattering cross-section as a function of surface-to-volume ratio for a prolate spheroid. The detuning is kept close to zero for all *S*/*V* values, which are obtained by varying the geometrical parameters of the particle. With increasing *S*/*V* the mode separation and the depth of the transparency dip is more apparent. The major semiaxis is varied in the range from 25 to 75 nm and the aspect ratio *a*/*b* from about 3.3 to 1.75. b) Dephasing parameters γ_{pl}, γ_0 and 2*g* as a function of *S*/*V*. c) Normalized scattering cross-section as a function of plasmon-exciton detuning, given as $\delta=\omega_{pl}-\omega_0$ for *S*/*V* kept at a value of 0.14. The detuning is achieved by embedding the particle into media with various artificially chosen dielectric functions ranging from n_3=1.10 to 1.40. Note the typical mode anti-crossing behavior, shown with two dotted lines serving as guides for the eye. d) Anti-crossing behavior plotted versus detuning. The dashed lines show positions of the plasmon and molecular resonances. Full squares – the upper and lower branches of the hybrid system.

In Figure S5c, scattering spectra of an individual core-shell particle is plotted versus the plasmon-exciton detuning, achieved by embedding the particle (a = 50 nm, $b = c$ = 17 nm)

into various optically homogeneous dielectrics with refractive index in the range of 1.10 – 1.40. A clear anti-crossing behavior, which is a characteristic of a strongly interacting system, is found. In panel d) this is shown more evidently. The graph shows the upper and lower branches of the coupled system, as well as the plasmon and exciton resonances (shown as dashed lines). The branches obviously do not cross and the splitting of about 70 meV is observed. The splitting is, however, weaker than the plasmon damping meaning that the system is not yet in the strong coupling regime in agreement with Fig. S5b.

Overall, the simulations presented in Fig. S5 qualitatively reproduce all essential features of experimental spectra shown in Figure 6 of the main text.

Supporting references:

1. Shegai, T.; Brian, B.; Miljković, V. D.; Käll, M. *ACS Nano* 2011, 5, (3), 2036-2041.
2. Shegai, T.; Chen, S.; Miljkovic, V. D.; Zengin, G.; Johansson, P.; Kall, M. *Nat Commun* 2011, 2, 481.
3. Shegai, T.; Miljković, V. D.; Bao, K.; Xu, H.; Nordlander, P.; Johansson, P.; Käll, M. *Nano Lett.* 2011, 11, (2), 706-711.
4. Johansson, P. *Phys. Rev. B* 2011, 83, 195408.
5. Ambjörnsson, T.; Mukhopadhyay, G.; Apell, S. P.; Käll, M. *Phys. Rev. B* 2006, 73, (8), 085412.
6. Meier, M.; Wokaun, A. *Opt. Lett.* 1983, 8, (11), 581-583.
7. Kelly, K. L.; Coronado, E.; Zhao, L. L.; Schatz, G. C. *J. Phys. Chem. B* 2002, 107, (3), 668-677.
8. Bohren, C. F.; Huffman, D. R., *Absorption and Scattering of Light by Small Particles*. John Wiley & Sons: New York, 1998.
9. Chen, H.; Shao, L.; Woo, K. C.; Wang, J.; Lin, H.-Q. *J. Phys. Chem. C* 2012, 116, (26), 14088-14095.
10. Johnson, P. B.; Christy, R. W. *Phys. Rev. B* 1972, 6, (12), 4370-4379.
